# Olfactory Receptor OR7A17 Expression Correlates with All-*Trans* Retinoic Acid (ATRA)-Induced Suppression of Proliferation in Human Keratinocyte Cells

**DOI:** 10.3390/ijms222212304

**Published:** 2021-11-14

**Authors:** Hyeyoun Kim, See-Hyoung Park, Sae Woong Oh, Kitae Kwon, Se Jung Park, Eunbi Yu, Seyoung Yang, Jung Yoen Park, Seoyoung Choi, Seoyoun Yang, Su Bin Han, Minkyung Song, Jae Youl Cho, Jongsung Lee

**Affiliations:** 1 Molecular Dermatology Laboratory, Department of Integrative Biotechnology, College of Biotechnology and Bioengineering, Sungkyunkwan University, Suwon City 16419, Gyunggi Do, Korea; yeon6389@skku.edu (H.K.); hanzeeoo@skku.edu (S.W.O.); wesdwe1@skku.edu (K.K.); tpwjd17@skku.edu (S.J.P.); yuebi95@skku.edu (E.Y.); yy1771@skku.edu (S.Y.); maria0502@skku.edu (J.Y.P.); csy2696@skku.edu (S.C.); chorim1004@skku.edu (S.Y.); subin816@skku.edu (S.B.H.); 2 Department of Bio and Chemical Engineering, Hongik University, Sejong City 30016, Korea; shpark74@hongik.ac.kr; 3 Integrative Research of T Cells Laboratory, Department of Integrative Biotechnology, College of Biotechnology and Bioengineering, Sungkyunkwan University, Suwon City 16419, Gyunggi Do, Korea; 4 Molecular Immunology Laboratory, Department of Integrative Biotechnology, College of Biotechnology and Bioengineering, Sungkyunkwan University, Suwon City 16419, Gyunggi Do, Korea

**Keywords:** olfactory receptor (OR), all-trans retinoic acid (ATRA), cell proliferation, calcium influx, G-protein-coupled receptor (GPCR), overexpression

## Abstract

Olfactory receptors (ORs), which belong to the G-protein-coupled receptor family, have been widely studied as ectopically expressed receptors in various human tissues, including the skin. However, the physiological functions of only a few OR types have been elucidated in skin cells. All-*trans* retinoic acid (ATRA) is a well-known medication for various skin diseases. However, many studies have shown that ATRA can have adverse effects, resulting from the suppression of cell proliferation. Here, we investigated the involvement of OR7A17 in the ATRA-induced suppression of human keratinocyte (HaCaT) proliferation. We demonstrated that OR7A17 is expressed in HaCaT keratinocytes, and its expression was downregulated by ATRA. The ATRA-induced downregulation of OR7A17 was attenuated via RAR α or RAR γ antagonist treatment, indicating that the effects of ATRA on OR7A17 expression were mediated through nuclear retinoic acid receptor signaling. Moreover, we found that the overexpression of OR7A17 induced the proliferation of HaCaT cells while counteracting the antiproliferative effect of ATRA. Mechanistically, OR7A17 overexpression reversed the ATRA-induced attenuation of Ca^2+^ entry. Our findings indicated that ATRA suppresses cell proliferation through the downregulation of OR7A17 via RAR α- and γ-mediated retinoid signaling. Taken together, OR7A17 is a potential therapeutic target for ameliorating the anti-proliferative effects of ATRA.

## 1. Introduction

Olfactory receptors (ORs)—members of the G-protein-coupled receptor (GPCR) superfamily—are predominantly expressed in the olfactory sensory neurons (OSNs) of the olfactory epithelium for smell detection and sensation [1]. While ORs were first described as being exclusively expressed in the nasal epithelium, various recent studies have reported their presence in nonolfactory tissues, including the skin [2,3]. A subset of ectopically expressed olfactory receptors, including OR2AT4, OR2A4/OR2A7, OR51B5, OR51E2, and OR10G7, have been identified and functionally characterized in human skin cells [4,5,6,7].

All-*trans* retinoic acid (ATRA) is a biologically active metabolite of vitamin A used for treating various skin diseases, such as psoriasis, acne, and ichthyosis. However, many studies have demonstrated the side-effects of ATRA, which include skin irritation, erythema, scaling, and dryness, resulting from disrupted epidermal barrier function due to dysregulated skin cell homeostasis [8,9,10,11,12,13]. The regulation of skin cell proliferation and differentiation is mediated via two specific nuclear receptors, namely RA receptors (RARs), which include α, β, and γ subtypes, and retinoid X receptors (RXRs). Upon binding to ATRA, RARs heterodimerize with RXRs, allowing the former to function as ligand-inducible transcriptional regulators. The heterodimers then bind to the RA response elements (RAREs) in the DNA, facilitating the further recruitment of transcriptional activators or repressors, which regulate target gene expression [14].

Besides skin disease, ATRA is widely used for the treatment of patients with acute promyelocytic leukemia (APL). ATRA induces cell cycle arrest and promotes the differentiation of APL cells into abnormal neutrophils [15]. Jung et al. demonstrated that ATRA influences ectopic OR expression on leukemia cells. They observed that ATRA-induced APL cell differentiation resulted in a decrease in OR expression via G9a histone methyltransferase (HMTase)- and lysine-specific histone demethylase 1 (LSD1)-mediated regulation. In addition, the knockdown of OR significantly inhibited the proliferation of APL cells [16].

While OR expression in skin cells and the antiproliferative effect of ATRA are well-established [17,18,19], the association between the two remains obscure. In the present study, we investigated the ectopic expression of OR—OR7A17—in human keratinocytes; the gene encoding OR7A17 has recently been identified as one of the genes associated with infectious skin traits in Holstein dairy cattle [20]. Further, we explored the functional association between OR7A17 and the antiproliferative effects of ATRA in HaCaT cells, a normal human keratinocyte cell line.

## 2. Results

### 2.1. ATRA Downregulated the Expression of OR7A17 in Human Keratinocytes

**Figure 1:**
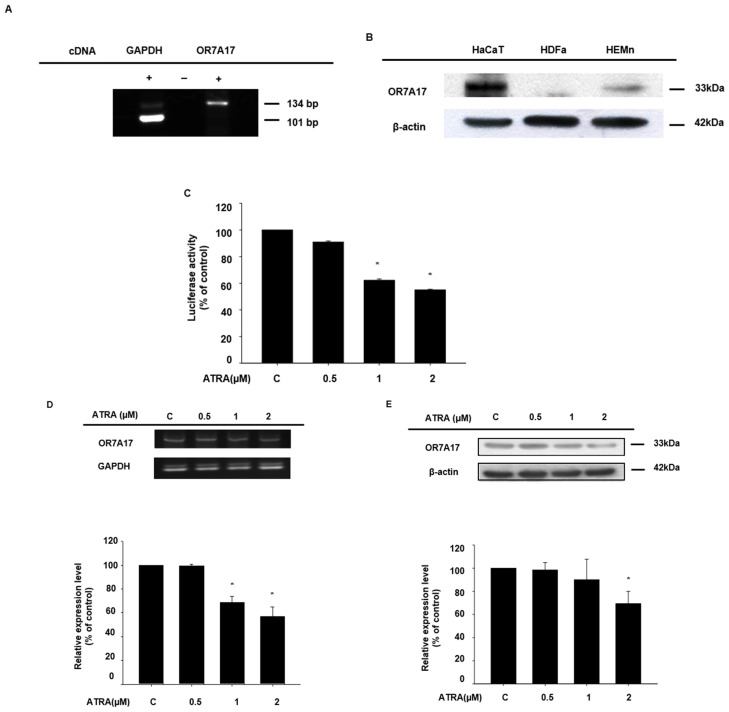
All-*trans* retinoic acid downregulates the expression of OR7A17. (**A**,**B**) The expression of OR7A17 in HaCaT cells was determined via reverse transcription quantitative PCR (RT-qPCR) (**A**) and Western blot analysis (**B**). In addition, OR7A17 expression was determined in human dermal fibroblasts and human epidermal melanocytes (**B**). HDFa: human dermal fibroblasts, HEMn: human epidermal melanocytes. (**C**) After ATRA treatment for 24 h, the cells were lysed and subjected to *OR7A17* luciferase reporter assays. (**D**) Changes in the mRNA levels of *OR7A17* in HaCaT cells were observed following treatment with ATRA for 24 h. (**E**) Changes in OR7A17 protein levels in HaCaT cells treated with ATRA for 24 h were determined via Western blot analysis. Data are presented as the mean ± SEM of at least three independent experiments. The statistical significance of differences between groups was evaluated via one-way analysis of variance (ANOVA), followed by Tukey’s multiple-comparison test, using the GraphPad Prism 5 software. * *p* < 0.05 vs. control.

To examine the effects of ATRA (Supplemental Figure S1), three concentrations, 0.5, 1, and 2 µM, were used in subsequent experiments, as it has already been demonstrated that the epidermal barrier function of HaCaT cells is disrupted following treatment with 1 µM of ATRA for 48 h [13]. We hypothesized that ATRA would suppress OR7A17 expression, based on a report by Jung et al. [16], who demonstrated that the expression of different ORs was repressed during the ATRA-induced differentiation of HL-60 leukemia cells. First, we determined the ectopic expression of OR7A17 in HaCaT keratinocytes via RT-qPCR (Figure 1A) and Western blot analysis (Figure 1B). We also found that OR7A17 was expressed in human epidermal melanocytes, but not in human dermal fibroblasts (Figure 1B). We conducted luciferase reporter assays to determine whether ATRA affects the *OR7A17* promoter activity. HaCaT cells transfected with the p*GL4.12-OR7A17* promoter were treated with ATRA for 24 h. As expected, the *OR7A17* promoter activity was significantly decreased by ATRA treatment, in a concentration-dependent manner (Figure 1C). This regulatory effect of ATRA was further evaluated by analyzing the mRNA (Figure 1D) and protein expression (Figure 1E) of OR7A17. The inhibitory effect of ATRA on both OR7A17 mRNA and protein expression was evident at a concentration of 2 µM. Taken together, these data indicated that OR7A17 expression in human keratinocytes is repressed by ATRA.

### 2.2. ATRA Suppresses the Proliferation of HaCaT Cells

**Figure 2:**
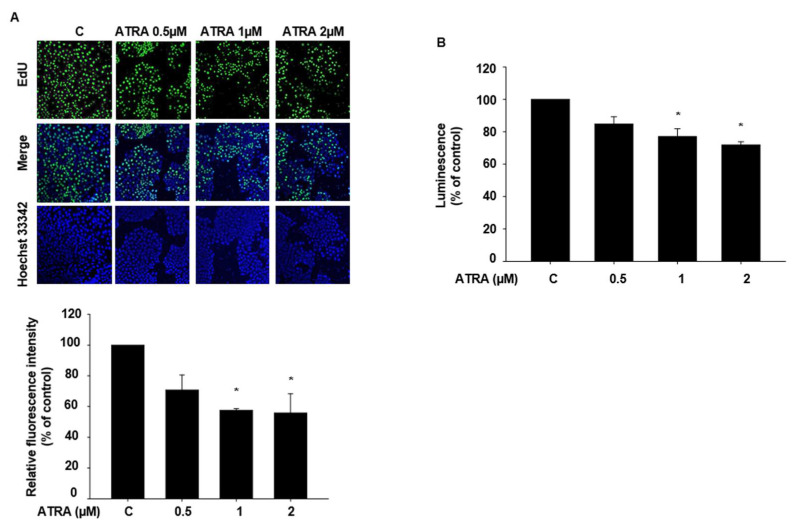
Effects of ATRA on the proliferation of HaCaT cells. (**A**) The effect of ATRA treatment for 48 h on HaCaT cell proliferation was determined via an EdU incorporation assay. The number of proliferating cells stained with EdU was normalized to that of Hoechst 3342-stained cells. (**B**) We confirmed the effect of ATRA on HaCaT cell proliferation via the CellTiter Glo^®^ 2.0 assay. The cells were incubated with ATRA for 48 h, and the change in luminescence was measured after the addition of CellTiter Glo^®^ 2.0 Reagent. Data are presented as the mean ± SEM of at least three independent experiments. The statistical significance of differences between groups was evaluated via one-way analysis of variance (ANOVA), followed by Tukey’s multiple-comparison test, using the GraphPad Prism 5 software. * *p* < 0.05 vs. control.

We conducted an EdU incorporation assay to examine the effect of ATRA on HaCaT cell proliferation. We first assessed whether a 24 h incubation with ATRA would influence cell proliferation. However, no significant change in proliferation was noted (data not shown). We then subjected the cells to 48 h of incubation with ATRA and observed that the proliferation of cells treated with 1 µM and 2 µM of ATRA was significantly decreased (Figure 2A). We confirmed these findings via the CellTiter^®^ Glo assay 2.0, which detects ATP in viable cells (Figure 2B).

### 2.3. OR7A17 Expression Is Regulated via RAR Signaling

**Figure 3:**
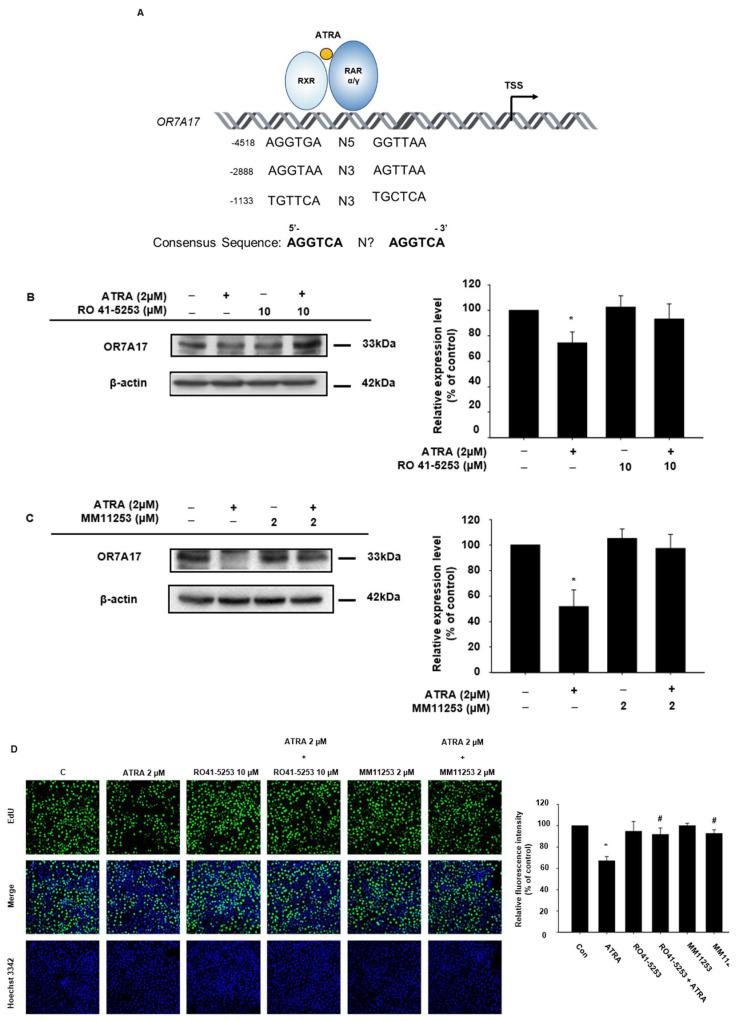
ATRA-induced downregulation of OR7A17 expression is mediated via RAR signaling. (**A**) A putative RARE sequence in the promoter region of the OR7A17 gene. (**B**,**C**) HaCaT cells were treated with 2 µM of ATRA and either 10 µM of RAR α antagonist (**B**) RO 41-5253 or 2 µM of RAR γ antagonist (**C**) MM11253 for 24 h. After 24 h of incubation, the cells were lysed, and Western blotting was performed to determine the protein levels of OR7A17. (**D**) The recovery of HaCaT proliferation following treatment with RO 41-5253/MM11253 and ATRA was observed via EdU incorporation assays. Data are presented as the mean ± SEM of more than three independent experiments. The statistical significance of differences between groups was evaluated via one-way analysis of variance (ANOVA), followed by Tukey’s multiple-comparison test, using the GraphPad Prism 5 software. * *p* < 0.05 vs. control, ^#^ *p* < 0.05 vs. ATRA-treated control.

In our previous experiments, the expression of *OR7A17* was downregulated by ATRA treatment. As shown in Figure 3A, we found that the promoter region of *OR7A17* contains a sequence similar to that of RARE where RXR/RAR complexes bind. This suggests that ATRA may downregulate the expression of *OR7A17* via the RARE. Therefore, we examined whether the expression of OR7A17 is regulated by RAR. As a previous study suggested that RAR *α* and RAR *γ* are the two main retinoic acid receptors expressed in human keratinocytes, whereas limited amounts for RAR β were detected in that study [17], we used RAR α antagonist RO41-5253 (Figure 3B) and RAR γ antagonist MM11253 (Figure 3C) to block the two receptors and determined the OR7A17 expression. We treated cells with ATRA for 24 h or ATRA plus either 10 µM of RO41-5253 or 2 µM of MM11253 for 24 h. In agreement with previous data, immunoblotting results indicated diminished OR7A17 protein levels when the cells were treated with ATRA alone. However, co-treatment with the antagonists recovered OR7A17 protein levels. In addition, reduced mRNA levels of *OR7A17* were attenuated by RO41-5253 and MM11253 (Supplemental Figure S2). These data indicated that the ATRA-induced repression of OR7A17 expression can be mediated by both RAR α and γ, suggesting that OR7A17 expression is dependent on retinoic acid signaling. Interestingly, as shown in Figure 3B,C, we observed two or three bands of OR7A17 protein in the Western blot. Although further research is needed, the appearance of several bands for OR7A17 might be attributed to post-translational modifications such as glycosylation, acetylation, and methylation [21,22,23]. Furthermore, to establish a connection between ATRA-regulated OR7A17 expression and ATRA antiproliferative effects, we subjected the cells treated with ATRA and RAR antagonists to EdU incorporation assays (Figure 3D). While treatment with antagonists alone did not affect the level of EdU intensity, when applied together with ATRA, the antiproliferative effect of the latter was significantly suppressed, as indicated by the recovered EdU intensity (Figure 3D). Therefore, we proved that the ATRA-induced changes in OR7A17 expression were mediated via RAR α and γ signaling. Further, OR7A17 levels played a critical role in HaCaT proliferation.

### 2.4. Overexpression of OR7A17 Interferes with the Suppressive Effect of ATRA

**Figure 4:**
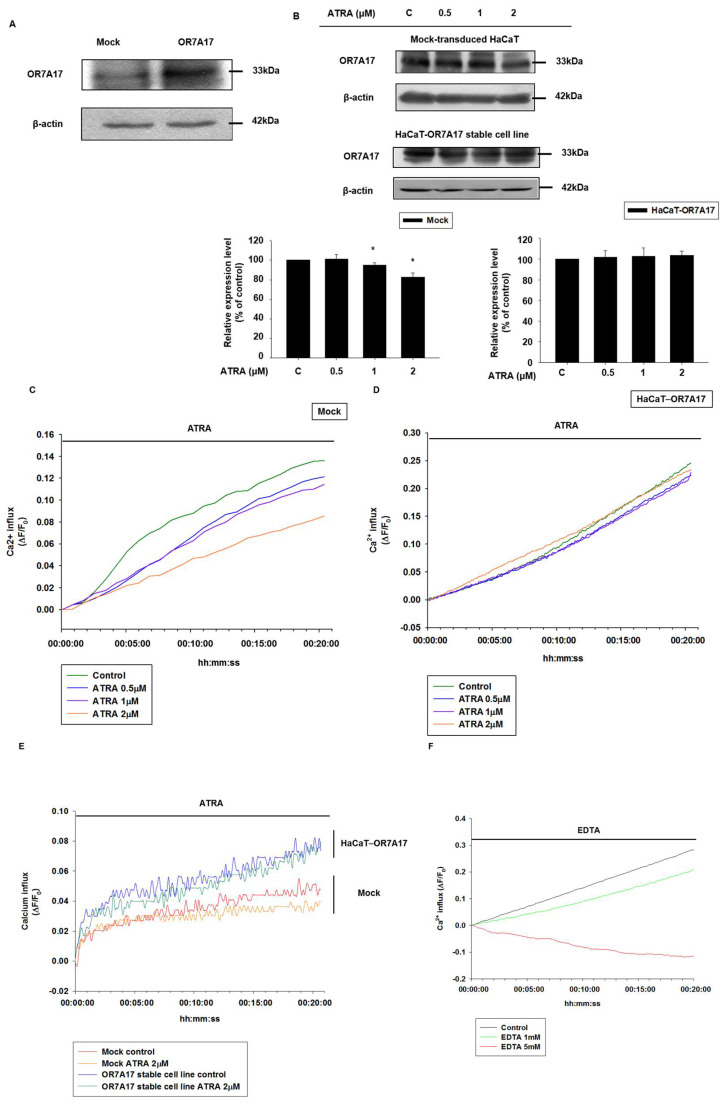

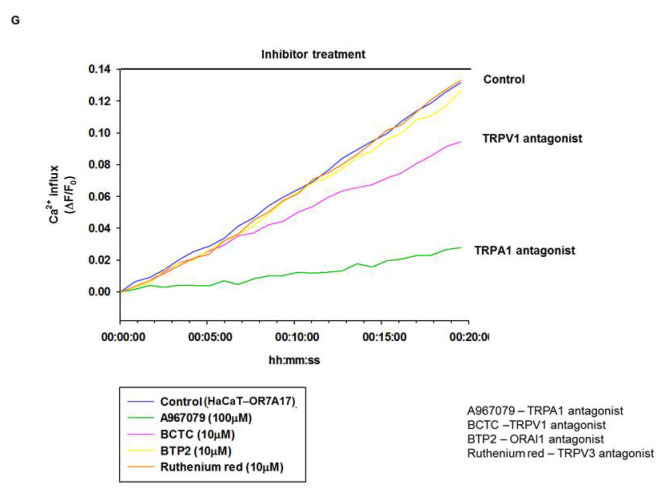
Overexpression of OR7A17 interferes with the suppressive effect of ATRA on Ca^2+^ influx. (**A**) HaCaT cells were transduced with either LvCMV-GFP (mock-transduced HaCaT cell line) or LvCMV-OR7A17 (HaCaT-OR7A17 stable cell line). The overexpression of OR7A17 in HaCaT−OR7A17 cells was validated via Western blot analysis. (**B**) HaCaT-OR7A17 cells and mock-transduced HaCaT cells were treated with ATRA for 24 h, and OR7A17 expression was determined via Western blot analysis. Data are presented as the mean ± SEM of more than three independent experiments. * *p* < 0.05 vs. control. Changes in Ca^2+^ influx after ATRA treatment were observed in (**C**) mock-transduced HaCaT cells and (**D**) HaCaT-OR7A17 cells. Cells were incubated with Fluo-4 NW Ca^2+^ reagent loading solution for 30 min at 37 °C and for another 30 min at room temperature. Fluorescence was measured immediately after ATRA treatment. The data presented are representative of at least three independent experiments. (**E**) Both mock-transduced HaCaT cells and HaCaT-OR7A17 cells were treated with ATRA and incubated for 24 h in an incubator at 5% CO_2_ and 37 °C. After 24 h of incubation, Fluo-4 NW Ca^2+^ reagent loading solution was added to the cells and they were incubated for 30 min at 37 °C and another 30 min at room temperature. Fluorescence was measured after the incubation. (**F**) Changes in Ca^2+^ influx after ethylenediaminetetraacetic acid (EDTA) treatment were observed in mock-transduced HaCaT cells. Cells were incubated with Fluo-4 NW Ca^2+^ reagent loading solution for 30 min at 37 °C and for another 30 min at room temperature. Fluorescence was measured immediately after EDTA treatment. The data presented are representative of at least three independent experiments. (**G**) HaCaT-OR7A17 cells were cultured and incubated for 24 h in a 5% CO_2_ incubator at 37 °C. After 24 h of incubation, Fluo-4 NW Ca^2+^ reagent loading solution was added and incubated for 30 min at 37 °C and for another 30 min at room temperature. The fluorescence was measured immediately after calcium channel inhibitor treatment. Data are representative of at least three independent experiments.

To further investigate the association of OR7A17 and the ATRA-induced suppression of proliferation, we induced *OR7A17* overexpression by using an LvCMV-OR7A17 vector, as described in the Materials and Methods. The successful transduction and overexpression of *OR7A17* in the HaCaT cell line was confirmed via Western blot analysis (Figure 4A). Next, we determined whether ATRA had any effect on OR7A17 levels under overexpression. Through Western blot analysis, we confirmed that ATRA did not affect OR7A17 levels in OR7A17-overexpressing HaCaT cells (Figure 4B). In contrast, ATRA regulated OR7A17 levels in the mock-transduced HaCaT cells (Figure 4B), as previously observed for normal HaCaT cells (Figure 1F).

OR function is dependent on Ca^2+^ channels and Ca^2+^ entry into the cell. We first examined whether ATRA treatment affected the intracellular level of Ca^2+^ in mock-transduced HaCaT cells. We conducted assays using the Fluo-4 NW Ca^2+^ assay kit, and the fluorescence intensity was measured for 30 min after ATRA treatment using Ca^2+^ dye reagent. As shown in Figure 4C, ATRA treatment reduced Ca^2+^ uptake in a concentration-dependent manner for 30 min. We conducted the same assay using HaCaT-OR7A17 cells, and we did not observe a significant decrease in the intracellular level of Ca^2+^. Rather, Ca^2+^ influx was maintained under treatment with ATRA at all concentrations tested (Figure 4D). Next, we investigated whether the change in Ca^2+^ influx levels was maintained after 24 h of incubation with ATRA. We treated cells with 2 µM of ATRA for 24 h and conducted an Ca^2+^ influx assay. Ca^2+^ influx remained low even after 24 h in mock-transduced HaCaT cells, while the HaCaT-OR7A17 stable cell line exhibited no significant reduction following ATRA treatment (Figure 4E). We also observed an increased basal level of Ca^2+^ influx in HaCaT-OR7A17 cells, indicating that the overexpression of *OR7A17* enhanced the activity of calcium channels and, consequently, Ca^2+^ influx (Figure 4E). In addition, using EDTA, we verified that these Fluo-4 signals were due to Ca^2+^ influx (Figure 4F). Furthermore, in the Ca^2+^ influx assay, we observed a constantly rising Ca^2+^ signal, even in the absence of ATRA application. Although the exact reason for this is not known, we can speculate that, similar to the NALCN (Na^+^ leak channel, nonselective) channel, it may be related to the leak conductance of Ca^2+^ channels during the resting state [24]. Collectively, these results suggested that ATRA leads to a reduction in Ca^2+^ influx in HaCaT cells, which can be reversed via *OR7A17* overexpression.

It has been established that various calcium channels operate downstream of ORs [6,25,26,27]. Therefore, we investigated potential calcium channels mediating OR7A17 function through the use of cation membrane channel inhibitors in HaCaT-OR7A17 cells. As shown in Figure 4G, while ORAI1 antagonist BTP2 and TRPV3 antagonist Ruthenium red had no effect on the level of Ca^2+^ influx, TRPV1 antagonist BCTC and TRPA1 antagonist A967079 suppressed Ca^2+^ influx. These observations suggested that OR7A17 downstream signaling may be mediated via TRPV1 or TRPA1. In addition, according to the suppressed Ca^2+^ influx by two inhibitors, the data suggest that OR7A17-mediated signaling is initially TRPV1-dependent and later TRPA1-dependent.

### 2.5. OR7A17 Overexpression Induces Proliferation of HaCaT Cells

**Figure 5:**
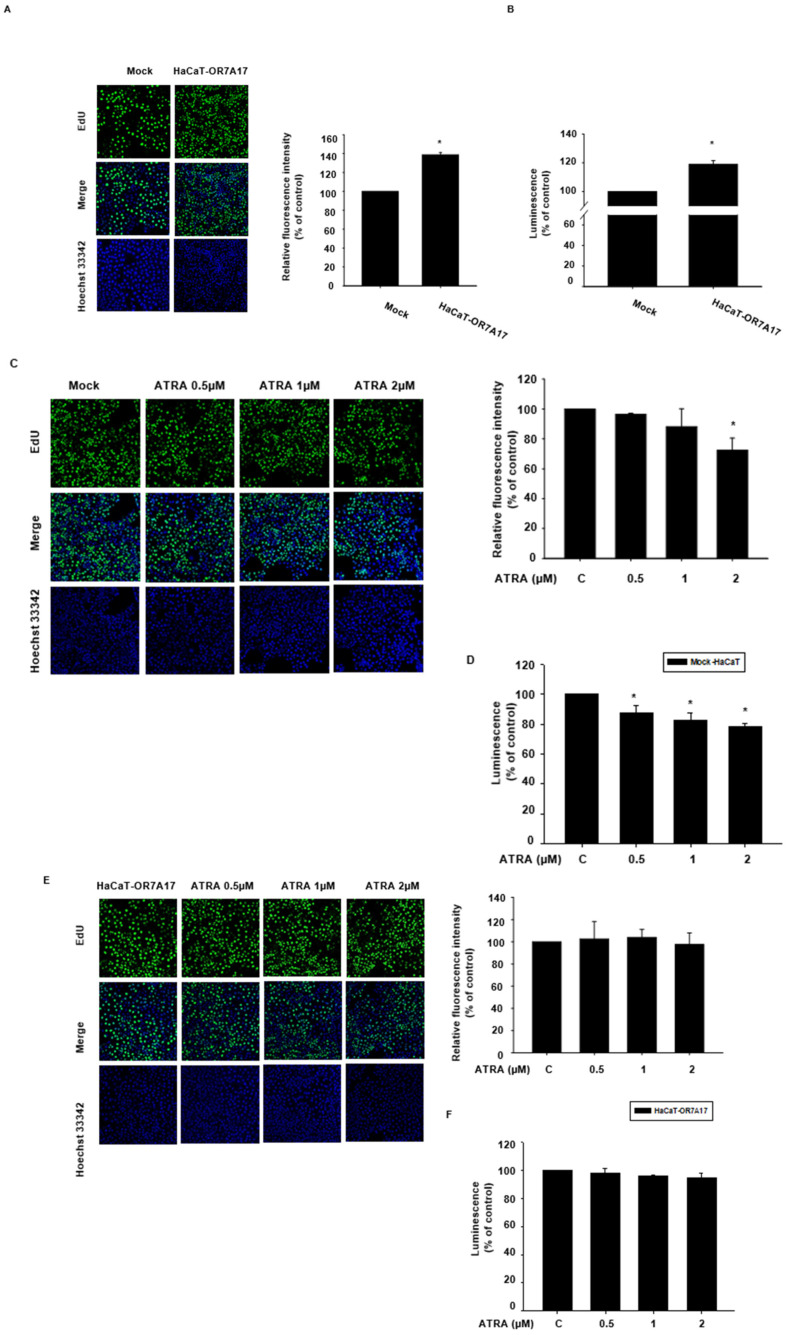
OR7A17 overexpression antagonizes the antiproliferative effect of ATRA in HaCaT cells. The proliferation-promoting effect of OR7A17 overexpression was observed via (**A**) EdU incorporation assays and (**B**) CellTiter^®^ Glo 2.0 assays. (**B**) The antiproliferative effect of ATRA was again confirmed in mock-transduced HaCaT cells via both (**C**) EdU incorporation assay and (**D**) CellTiter^®^ Glo 2.0 assay. HaCaT-OR7A17 cells were treated with ATRA for 24 h as in previous experiments. (**E**) EdU incorporation assay and (**F**) CellTiter^®^ Glo 2.0 assay were carried out, and no significant change in proliferation was observed in OR7A17-overexpressing cells. Data are presented as the mean ± SEM of at least three different independent experiments. The statistical significance of differences between groups was evaluated via one-way analysis of variance (ANOVA), followed by Tukey’s multiple-comparison test, using the GraphPad Prism 5 software. * *p* < 0.05 vs. control.

Our findings indicated that OR7A17 is involved in the proliferation of human keratinocytes. Therefore, we investigated whether the overexpression of *OR7A17* would be sufficient to induce the proliferation of HaCaT cells. To this end, we conducted two different proliferation assays, namely an EdU incorporation assay and a CellTiter^®^ Glo assay 2.0. As expected, HaCaT cells transduced with LvCMV-OR7A17 exhibited a significantly greater proliferation than that by the mock-transduced cells, as indicated by both the EdU incorporation assay (Figure 5A) and CellTiter^®^ Glo 2.0 assay (Figure 5B). In order to assess the antiproliferative effect of ATRA on HaCaT-OR7A17 cells, the same proliferation assays were performed with HaCaT-OR7A17 and mock-transduced cells. ATRA was added to the cells, and they were incubated for 48 h at 37 °C in an incubator at 5% CO_2_. ATRA treatment suppressed the proliferation of mock-transduced HaCaT cells as indicated by both the EdU incorporation assay (Figure 5C) and CellTiter^®^ Glo assay 2.0 (Figure 5D). In contrast, HaCaT-OR7A17 proliferation was not affected by ATRA (Figure 5E,F). Taken together, these data indicated that *OR7A17* overexpression induced the proliferation of HaCaT cells, counteracting the antiproliferative effect of ATRA.

## 3. Discussion

The expression of ORs in various nonolfactory tissues has been investigated by researchers since the early 1990s, following the first report of OR gene transcripts in mammalian germ cells [28]. In the last few years, an increasing number of studies have characterized the functions of ORs ectopically expressed in the human skin, reporting their involvement in the proliferation, differentiation, migration, and re-epithelialization of skin cells [4,5,6,7,29]. While the OR gene family is known to constitute the largest group of *GPCR* genes [30], only a few types of ORs have been studied. Further, most ORs are currently classified as orphan receptors, as their activators have not yet been identified. Therefore, exploring OR expression and function in skin cells is of considerable interest.

In this study, we confirmed the expression of OR7A17 in HaCaT cells using RT-qPCR and Western blotting. As an orphan receptor, OR7A17 has not been functionally characterized in human skin cells. Thus, we sought to elucidate OR7A17 expression and function in keratinocytes.

ATRA is a signaling molecule derived from vitamin A, which regulates the proliferation, differentiation, and death of various cell types in humans [31]. More specifically, it impairs epidermal barrier function by altering the expression of proteins involved in cell proliferation and differentiation [13]. Memezawa et al. (2007) previously demonstrated that the crosstalk between canonical Wnt signaling and retinoid signaling is behind the antiproliferative potency of ATRA [17]. Further, they determined that inhibitor of DNA binding 2 (Id2), a protein that is responsible for regulating the cell cycle progression [32,33], was downregulated in HaCaT cells subjected to ATRA treatment [17]. In the current study, we found that OR7A17 is also downregulated by ATRA at both the gene and protein level via retinoid signaling, specifically the RAR α and γ subtypes. It is well-established that retinoic acid signaling can either activate or repress gene expression. Fibroblast growth factor 8 (*Fgf8*) is one of the genes repressed by retinoic acid and its co-regulators [34]. Therefore, it is plausible to consider *OR7A17* as a potential target gene of retinoid signaling. We confirmed the involvement of *OR7A17* in the antiproliferative effect of ATRA by overexpressing it. Indeed, the overexpression of *OR7A17* induced HaCaT cell proliferation. It has been reported that different types of ORs, such as OR2A4/7 and OR2AT4, are involved in human keratinocyte proliferation upon activation via their specific odorants [4,5]. However, our current findings suggest that human keratinocyte proliferation was affected by OR7A17 expression alone, in the absence of receptor activation by its specific agonist.

In human skin cells, Ca^2+^ levels increase from the basal layer toward the outer layer of the epidermis [35]. This gradient facilitates the regulation of various cellular processes in keratinocytes, including cell survival, proliferation, motility, apoptosis, and differentiation [36]. Calcium ion levels are highly dynamic, as ions are released from intracellular stores or enter from extracellular sources, driving Ca^2+^ signaling in cells [37,38,39]. Ca^2+^ homeostasis is associated with the control of cell cycle progression and, inevitably, proliferation [40]. Thus, we sought to determine whether ATRA treatment alters the intracellular Ca^2+^ levels in HaCaT cells as a mechanism underlying its antiproliferative effect. ATRA treatment reduced the Ca^2+^ influx in a concentration-dependent manner in HaCaT cells, while no change was observed in HaCaT-OR7A17 cells. Similarly, Bonnefond et al. demonstrated that carboxyamidotriazole, a Ca^2+^ channel blocker, exerted an antiproliferative effect in several normal and transformed cell types [41,42]. Based on the current findings, we were able to conclude that OR7A17 mediated ATRA-induced changes in Ca^2+^ signaling and, consequently, its antiproliferative effect in HaCaT cells. However, we cannot exclude the possibility that other ATRA/RXR-RAR-induced proteins/pathways might be responsible for Ca^2+^ signaling in HaCaT cells.

Various types of Ca^2+^ channels, such as the transient receptor potential vanilloid type 6 (TRPV6) channel, transient receptor potential melastatin (TRPM) channel, cyclic nucleotide-gated ion channel (CNG) channel, and calcium release-activated calcium channel protein 1 (ORAI1), have been demonstrated to induce Ca^2+^ signaling responses following the activation of specific OR types [6,25,26,27]. While we suggested that Ca^2+^ channels TRPV1 and TRPA1 may be associated with the function of OR7A17 based on channel blocker experiments, further investigation is still necessary.

## 4. Materials and Methods

### 4.1. Cell Culture

Human immortalized keratinocyte cell line HaCaT cells (American Type Culture Collections, Manassas, VA, USA) were cultured and maintained in Dulbecco’s modified Eagle’s medium (DMEM). DMEM was supplemented with 10% fetal bovine serum (FBS) and 1% antibiotics (penicillin/streptomycin). The cells were maintained in 5% CO_2_ humidified air at 37 °C. HaCaT-OR7A17 cells and mock-transduced HaCaT cells were maintained in DMEM supplemented with 10% FBS, 1% antibiotics, and 0.1% puromycin under the same conditions.

### 4.2. ATRA Treatment

ATRA was purchased from Sigma-Aldrich, St. Louis, MO, USA. A stock solution (1000× stock) was prepared in DMSO and stored in the dark at −20 °C until use. To obtain the final concentrations (0.5 µM to 20 µM), ATRA was further diluted with DMEM.

### 4.3. Fluo-4 Ca^2+^ Influx Assay

The Ca^2+^ influx assay was conducted using a Fluo-4 NW Ca^2+^ assay kit (cat no. F36206, Invitrogen, Waltham, MA, USA). Cells were seeded at a density of 1 × 10^4^ cells/well in 96-well black wall/white bottom microplates and incubated for 24 h. After 24 h, the growth medium was removed, and 1× Fluo-4 NW Ca^2+^ reagent loading solution was directly added to each well. The cells were incubated with the dye loading solution at 37 °C for 30 min, followed by incubation at room temperature for an additional 30 min in the dark. After incubation, fluorescence was measured using a microplate reader (Synergy HTX Multi-Mode Reader, Biotek, Winooski, VT, USA) at excitation/emission wavelengths of 494/516 nm.

### 4.4. Luciferase Reporter Assay and β-Galactosidase Activity Assay

Luciferase reporter assays and β-galactosidase activity assays were performed to determine the gene promoter activity. The cells were seeded in 6-well plates, incubated at 37 °C for 24 h, and co-transfected with 1 µg of *OR7A17* promoter-luciferase reporter and 1 µg of β-galactosidase vector (Promega Corporation, Madison, WI, USA) using 5 µg of polyethylenimine (Sigma-Aldrich, St. Louis, MO, USA). After 4 h of transfection, the growth medium was replaced with fresh medium, and the cells were incubated in a CO_2_ incubator overnight. Next, growth medium containing ATRA was replaced again, and the cells were incubated for 24 h. Cells were harvested with PBS and lysed with the reporter lysis buffer (Promega Corporation, Madison, WI, USA), and the β-galactosidase assay was carried out using the *β*-galactosidase enzyme assay system (Promega Corporation, Madison, WI, USA). The lysed cells were centrifuged, and the supernatants were transferred into 96-well plates for the β-galactosidase assay. The cells were incubated at 37 °C until color development was observed, and the color development was stopped by adding 1 M of sodium carbonate to the wells. The absorbance of each well was measured at 420 nm for β-galactosidase activity quantification. A luciferase activity assay system (Promega Corporation, Madison, WI, USA) was used to quantify the *OR7A17* promoter-luciferase reporter activity. The supernatant from the lysed cells was transferred into white 96-well plates. The substrate and buffer provided in the luciferase activity assay system (Promega Corporation, Madison, WI, USA) were added to the samples in the wells, and luminescence was measured using a microplate reader (Synergy HTX Multi-Mode Reader, Biotek, Winooski, VT, USA). The promoter activity of *OR7A17* was expressed as the ratio of *OR7A17*-dependent firefly luciferase activity to β-galactosidase activity.

### 4.5. Western Blotting Analysis

Cells were cultured in 60 mm cell plates, harvested, and centrifuged at 16,200× *g*. The supernatant was discarded, and the cell pellets were lysed with RIPA lysis buffer (25 mM of Tris-HCl (pH 7.6), 150 mM of NaCl, 1% NP-40, 1% sodium deoxycholate, and 0.1% SDS) (Thermo Fisher Scientific, Waltham, MA, USA) containing Halt protease and a phosphatase inhibitor cocktail (Thermo Fisher Scientific, Waltham, MA, USA). The cells with RIPA lysis buffer were then centrifuged at 21,055× *g* for 50 min to extract proteins. The supernatant was transferred to Eppendorf safe-lock tubes and was stored at −70 °C until use. The 40 µg of extracted proteins was loaded per lane and separated via 12% SDS electrophoresis and transferred onto nylon membranes. The membranes were blocked with 3% bovine serum albumin (BSA) for 1 h and then incubated overnight with primary antibodies at 4 °C. The membranes were washed at least three times with Tris-buffered saline (TBS) containing Tween 20 and then incubated with the secondary antibodies for at least 1 h at room temperature. The blots were visualized using ECL Western blotting reagents.

### 4.6. Antibodies

The following primary antibodies, which were diluted to 1:500 and 1:1000, respectively, were used: rabbit polyclonal antibody against OR7A17 (cat. no PA5-71202, Invitrogen, Waltham, MA, USA); mouse monoclonal antibody against β-actin (cat. no A5316, Sigma-Aldrich, St. Louis, MO, USA). The following secondary antibodies, which were diluted to 1:4000, were used: anti-mouse IgG (cat no. A9044, Sigma-Aldrich, St. Louis, MO, USA); anti-rabbit IgG (cat no. A0545, Sigma-Aldrich, St. Louis, MO, USA).

### 4.7. Reverse Transcription qPCR (RT-qPCR)

Total RNA was isolated from cells using the TRI reagent^®^ as per the manufacturer’s instructions. Two micrograms of isolated total RNA was reverse-transcribed into cDNA using the TOPscript™ RT DryMIX (Enzynomics. Deajeon, South Korea) as per the manufacturer’s instructions. cDNA was amplified using specific primers against human *OR7A17* cDNA and *GAPDH* cDNA. The reaction parameters for PCR and PCR primer information are described in Supplementary Table S1. The amplified PCR products were subjected to electrophoresis on 3% agarose gels and visualized under UV radiation.

### 4.8. EdU Incorporation Assay

EdU incorporation assays for cell proliferation analysis were carried out using the Click-iT™ EdU cell proliferation kit for imaging (cat. no *A10044*, Invitrogen, Waltham, MA, USA) according to the manufacturer’s instructions. Approximately 10 µM of EdU was added to cells grown on glass coverslips, and the coverslips were incubated for 12 h. The cells were washed three times with PBS after every step, fixed using 4% paraformaldehyde in PBS for 15 min, and permeabilized in 0.1% Triton X-100 and 0.01% Tween 20 for 20 min at room temperature. Thereafter, the cells were blocked with 3% BSA in PBS for 1 h. After blocking, the cells were stained via incubation for 30 min with the Click-iT^®^ reaction cocktail in the dark as per the manufacturer’s instructions. After washing with PBS three times, the cells were counterstained with Hoechst 33342 (Invitrogen, Waltham, MA, USA). Finally, the cells were mounted on glass slides with PBS and observed under an LSM 700 laser-scanning confocal microscope (Zeiss, Jena, Germany) with a C-Apochromat 10× objective. The images were captured under the same laser intensity, and the mean intensity of fluorescence signals was measured. Images were analyzed using ZEN 2012 Blue (Zeiss, Jena, Germany) and ImageJ software (National Institutes of Health, Bethesda, MD, USA).

### 4.9. CellTiter Glo^®^ 2.0 Assay for Cell Proliferation Analysis

Cells (5 × 10^3^ cells) were seeded in an opaque-walled multi-well plate with culture medium. After incubating the cells at room temperature for 24 h, ATRA was added and the plate was incubated for 48 h in an incubator at 5% CO_2_ and 37 °C. After 48 h of ATRA treatment, the cells were kept at room temperature for 30 min. CellTiter Glo^®^ 2.0 reagent (Promega Corporation Madison, WI, USA) was added at a volume equal to that of the cell culture medium in each well. Plates were mixed for 2 min on a shaker and incubated at room temperature for another 10 min. The luminescence was recorded.

### 4.10. Lentiviral Transduction of HaCaT Cells

To establish a stable OR7A17 cell line, cells (1 × 10^5^ cells/well) were first plated in 12-well plates along with Lentifect™ purified lentiviral particles (GeneCopoeia, MD, USA) encoding *OR7A17* (multiplicity of infection (MOI) of 20) in DMEM containing 8 µg/mL of polybrene. After overnight incubation at 37 °C and 5% CO_2_, the medium was changed with DMEM containing 1 µg/mL of puromycin for selection. The medium was replaced with fresh puromycin-containing medium every 3–4 days until only resistant colonies were identified. The transduced keratinocytes were grown to almost confluence, trypsinized, and added into new plates.

### 4.11. Statistical Analysis

Data are expressed as the mean ± standard error of the mean (SEM) of at least three independent experiments. Differences between groups were evaluated via one-way analysis of variance (ANOVA), followed by Tukey’s multiple-comparison test, using GraphPad Prism (5.0) (GraphPad, La Jolla, CA, USA). Differences were considered significant when *p* < 0.05.

## 5. Conclusions

**Figure 6:**
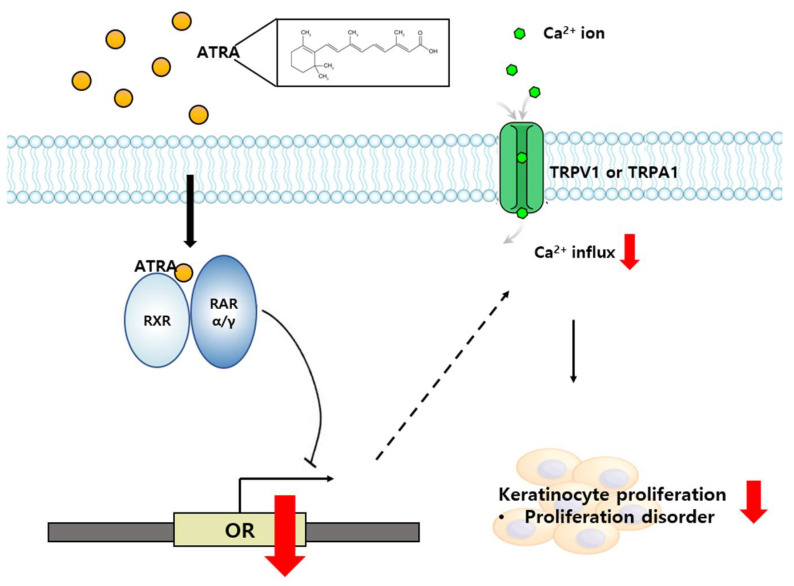
Schematic of OR transcriptional regulation mediating the antiproliferative effects of ATRA on human keratinocyte. OR: OR7A17.

To summarize, we demonstrated the involvement of OR7A17 in the ATRA-induced suppression of HaCaT cell proliferation. ATRA downregulated OR7A17 expression through RAR α and γ signaling, and the suppression of OR7A17 expression decreased Ca^2+^ influx, eventually leading to reduced cell proliferation. OR7A17-mediated signaling is described in Figure 6. In conclusion, we determined the physiological function of OR7A17, which has not been previously explored in human keratinocytes. Though further investigation into the exact mechanism of OR7A17 and its role in proliferation is necessary, our findings highlight OR7A17 as a potential therapeutic target for counteracting the antiproliferative effects of ATRA on keratinocytes.

## Data Availability

The data used to support the findings of this study are available from the corresponding author upon request.

## Supplementary Materials

The following are available online at https://www.mdpi.com/article/10.3390/ijms222212304/s1.
