# Supplementary material for: Olfactory Receptor OR7A17 Expression Correlates with All-Trans Retinoic Acid (ATRA)-Induced Suppression of Proliferation in Human Keratinocyte Cells
Source: Int J Mol Sci. 2021 Nov 14;22(22):12304. doi: 10.3390/ijms222212304 (PMC8623719; doi:10.3390/ijms222212304)
Supplement: Supplementary file 1 [file ijms-22-12304-s001.zip › ijms-1428958-supplementary.pdf]

Supplementary Table S1. The reaction parameters for PCR and PCR primer information

PCR condition			
**Step**	**Temperature (˚C)**	**Time**	**No. of cycles**
Initial denaturation	94	3 min	1
Denaturation	94	15 sec	
Annealing	60	30 sec	40
Elongation	72	20 sec	
Final elongation	72	5 min	1
Storage	8		

Gene name	Primer sequence (5' – 3')	Product size (bp)
OR7A17	Forward – TGG AAC CAG AGA ATG ACA CAG G<br>Reverse - GAT GAG CAG ATT CCC GAG CA	134
GAPDH	Forward – ACA ACT TTG GTA TCG TGG AAG G<br>Reverse - GCC ATC ACG CCA CAG TTT C	101
GAPDH2<br>(supplementary data)	Forward –GGA GCC AGA TCC CTC CAA AAT<br>Reverse – GGC TGT TGT CAT ACT TCT CAT GG	197

Supplemental Fig. S1

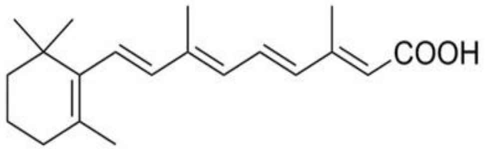

Supplemental fig. S1. Chemical structure of All-*trans* retinoic acid (ATRA).

Supplemental Fig. S2

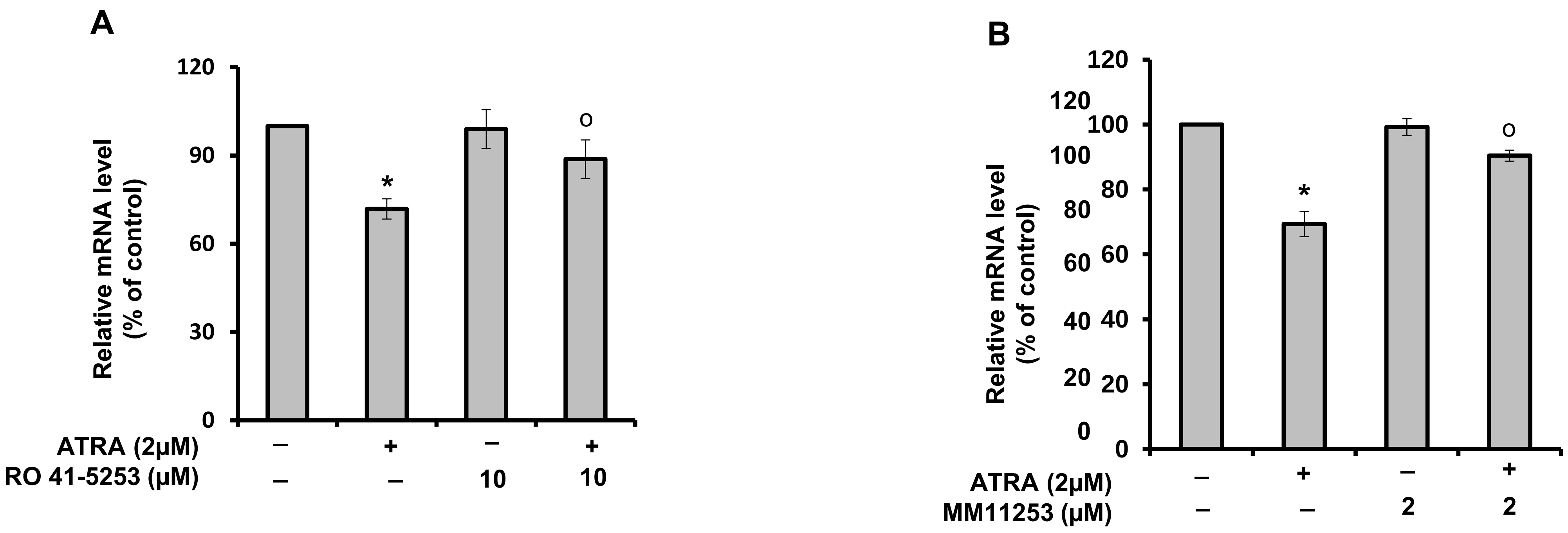

Supplemental Fig. S2. Effect of RO41-5253 and MM11253 on the reduced mRNA levels of OR7A17 induced by ATRA treatment. ,HaCaT cells were treated with 2 μM ATRA and either 10 μM RAR α antagonist (A) RO 41-5253 or 2 μM RAR γ antagonist (B) MM11253 for 24 h. After 24 h of incubation, the cells were lysed, and real-time PCR analysis was performed to determine the mRNA levels of OR7A17. Data are presented as the mean ± SEM of more than three independent experiments. * $p < 0.05$ vs. control, ° $p < 0.05$ vs. ATRA-treated group.
